# Editorial: Development and Potential Mechanisms of Low Molecular Weight Drugs for Cancer Immunotherapy

**DOI:** 10.3389/fimmu.2022.920442

**Published:** 2022-05-17

**Authors:** Yanfeng Gao, Xuanming Yang, Bin Zhang

**Affiliations:** ^1^ School of Pharmaceutical Sciences (Shenzhen), Sun Yat-sen University, Shenzhen, China; ^2^ School of Life Science and Biotechnology, Shanghai Jiao Tong University, Shanghai, China; ^3^ Department of Medicine-Division of Hematology/Oncology, Northwestern University Feinberg School of Medicine, Chicago, IL, United States

**Keywords:** cancer immunotherapy, innate immunity, immune checkpoint, drug repositioning, small molecular inhibitor

Cancer immunotherapy has gained great progress during the last decade. More than ten PD-1/PD-L1 antibodies were approved globally. However, the response rate in certain types of cancer is still need to be improved. On the other hand, low molecular weight therapeutics, such as proteins, peptides and small molecules, have gained more attention recently with their advantages including greater penetration, oral bioavailability, easy modification and fine control of bioavailability, meanwhile avoiding some severe immune related adverse events associated with antibodies. Therefore, they are considered as the next-generation of cancer immunotherapy candidates. However, their application and regulation role in cancer immunotherapy are largely needed to be explored.

The present Research Topic “Development and Potential Mechanisms of Low Molecular Weight Drugs for Cancer Immunotherapy” accepted twelve articles including seven research articles, four review articles and one case report. These articles covered a broad area including small molecule agents targeting immune checkpoint PD-1 and IDO, anti-PD-1 development and novel combination strategies, repositioning of vitamin C and curcumin derivates, cGAS inhibitor and bacteria to modulate the innate immunity.

Up to now, seven PD-1/PD-L1 antibody entities has been approved FDA. Toripalimab is the first PD-1 antibody approved by CFDA for the treatment of melanoma (second-line) in December, 2018. Zhang et al. described the development and clinical application of Toripalimab in China. Aside from the long-term survival benefits of Toripalimab monotherapy in Chinese melanoma patients, the combination with axitinib also exhibited an impressive clinical outcome. Meanwhile, Yang et al. reported a 58-year-old female patient with renal cell carcinoma resistant to both anti-PD-1 monotherapy and standard-dose axitinib. Interestingly, after the low dose of axitinib was given, the regulatory T cells decreased gradually with the tumor regressed. These results suggested that synergistic effects can be achieved by the combination of targeted therapy and anti-PD-1, and the treatment timing and dosage is critical for combinational efficacy. On the other hand, a series of chemotherapeutic agents are reported to induce the apoptosis and autophagy of tumor cells, which could lead to immunogenic cell death (ICD). But the high dosage and inappropriate timing would impair the antitumor immune response. Yuan et al. reported that a low-dose chemotherapy/autophagy enhancing regimen (CAER) could induce the death of tumor cells with higher levels of autophagy. These results further emphasized the importance of precise combination of these antitumor strategies.

Although anti-PD (PD-1/PD-L1) antibodies have made great clinical success, the therapeutic resistance and immune-related adverse events are still concerns. Moreover, the poor ability of solid tumor penetration and injection-only administration property promote the development of low molecular weight therapeutics. Wang et al. reported the discovery of a PD-1/PD-L1 inhibitor PDI-1 with antitumor activity. Sasikumar and Ramachandra summarized the small molecules targeting PD-1 for cancer immunotherapy. The structure, function and mechanism were analyzed. More importantly, the ongoing clinical trials of the small molecules and experience learned were also discussed. The development of inhibitors targeting other immune checkpoints is also thought to be helpful to improve the anti-PD therapeutic efficacy. Unfortunately, the IDO1 inhibitor epacadostat failed in phase III clinical trials. Shao et al. discovered that the IDO1 metabolite kynurenine could restrict the antitumor effects of CAR-T therapy in solid tumor. Therefore, IDO1 inhibitor was used as a combination by nanomaterial based delivery system to improve its therapeutic efficacy.

Drug repositioning comes from the idea that we can discover the novel mechanisms or indications of the approved drugs and natural products with identified toxicity. Curcumin is a well-known anticancer natural product. Wang et al. reported a novel curcumin derivative to inhibit colon cancer cells through changing the mitochondrial membrane potential and inducing endoplasmic reticulum (ER)-stress. Sohail et al. summarized the recent progress of a lipophilic analog of curcumin (dimethoxycurcumin) which maintains the anticancer potency but improves the systematic bioavailability. Similar as curcumin to regulate the production of Reactive oxygen species (ROS), vitamin C elicits antitumor activity through oxidant and epigenetic mechanisms. Kouakanou et al. summarized the recent progress of vitamin C to modulate the function of immune cells, and to improve their antitumor effects, such as adoptive cellular therapies.

Modulation of the signaling pathways of innate immune system can amplify the antitumor response or relieve the immune-related side effects, such as Toll-like receptors (TLRs) and cGAS-STING pathways. The components from bacteria have been considered as the powerful immune stimulator historically in clinic. Yang et al. discovered that the engineered anaerobic *Salmonella typhimurium* strain YB1 could initiate the phagocytosis function of macrophages and neutrophils and thus inhibit tumor growth and metastasis. Yu et al. established an intratumoral injection model by using PcrV protein from *Pseudomonas aeruginosa* and found tumor growth suppression effects. PcrV protein could activate the TLR4/MyD88 pathway and thus induce the innate immune response, such as potentiate the M1 polarization of macrophage. Chu et al. discovered a natural monoterpenoid compound perillaldehyde (PAH), derived from *Perilla frutescens*, could elicit cGAS inhibitory effects and have the potential to treat autoimmune syndrome. These studies open a window to develop novel strategies to strengthen the cancer immunotherapy or control the side effects.

Although no low molecular weight immunotherapeutic drugs have been approved up to now, more and more encouraging results have been reported from clinical trials. We believe that this Research Topic will help us to understand the development of next-generation of therapeutic agents for cancer immunotherapy in “post anti-PD era”.

## Author Contributions

YG wrote the manuscript. XY and BZ revised the text. All authors contributed to the article and approved the submitted version.

## Funding

This work was supported by grants from the National Natural Science Foundation of China (No. U20A20369).

## Conflict of Interest

The authors declare that the research was conducted in the absence of any commercial or financial relationships that could be construed as a potential conflict of interest.

## Publisher’s Note

All claims expressed in this article are solely those of the authors and do not necessarily represent those of their affiliated organizations, or those of the publisher, the editors and the reviewers. Any product that may be evaluated in this article, or claim that may be made by its manufacturer, is not guaranteed or endorsed by the publisher.

